# Effects of the Manufacturing Process on the Reliability of the Multilayer Structure in MetalMUMPs Actuators: Residual Stresses and Variation of Design Parameters

**DOI:** 10.3390/mi8120348

**Published:** 2017-11-29

**Authors:** Jianbin Guo, Jinling Wang, Shengkui Zeng, Vadim V. Silberschmidt, Yongguang Shen

**Affiliations:** 1 School of Reliability and Systems Engineering, Beihang University, Beijing 100191, China; guojianbin@buaa.edu.cn (J.G.); shenyongguang@buaa.edu.cn (Y.S.); 2 School of Electrical and Electionic Engieering, Zibo Vacational Insititute, Zibo 255314, China; wangjinling@buaa.edu.cn; 3 Wolfson Mechanical and Manufacturing Engineering, Loughborough University, Leicestershire LE11 3TU, UK; V.Silberschmidt@lboro.ac.uk

**Keywords:** micro electromechanical systems (MEMS), residual stresses, subdomain method, manufacturing process, multilayer structure

## Abstract

Potential problems induced by the multilayered manufacturing process pose a serious threat to the long-term reliability of MEMSCAP^®^ actuators under in-service thermal cycling. Damage would initiate and propagate in different material layers because of a large mismatch of their thermal expansions. In this research, residual stresses and variations of design parameters induced by metal multi-user micro electromechanical system processes (MetalMUMPs) were examined to evaluate their effects on the thermal fatigue lifetime of the multilayer structure and, thus, to improve MEMSCAP^®^ design. Since testing in such micro internal structure is difficult to conduct and traditional testing schemes are destructive, a numerical subdomain method based on a finite element technique was employed. Thermomechanical deformation from metal to insulator layers under in-service temperature cycling (obtained from the multiphysics model of the entire actuator, which was validated by experimental and specified analytical solutions) was accurately estimated to define failures with a significant efficiency and feasibility. Simulation results showed that critical failure modes included interface delamination, plastic deformation, micro cracking, and thermal fatigue, similarly to what was concluded in the MEMSCAP^®^ technical report.

## 1. Introduction

Multilayer structures have been widely used in various micro- and nanoscale components because of their better electrical continuity and thermal insulation. Alongside these advantages, it has also been demonstrated that multilayer interfaces are one of the most common sites of potential failures, since they often have weak bonding (low resistance to fracture) and are sites of large stress concentration (high driving force for fracture) as a result of the deformation mismatch of dissimilar materials [1].

Recently, several scholars have realized the reliability problems of multilayer structures and have attempted to analyse their failure behaviours. In [2,3,4], the scholars examined the interface delamination failure in various micro electromechanical systems (MEMS) devices. Sumigawa et al. studied the crack initiation at a Si–Cu–SiN–Au interface caused by stress concentration [1]. Zhu et al. [5] also investigated the crack initiation strain of a Cu–Ni multilayer on a polyimide substrate. They explored the critical value of strain for increasing crack density. In addition, Köckritz et al. [6] studied the modification of the layered structure according to the requirements of each layer of material to improve its lifetime and performance. Yan et al. [7] studied the effect of the environmental factors temperature and humidity on the bending fatigue strength of a Cu–Si interface. The thermal fatigue lifetime of a Si–Ceramic–Cu interface was predicted with the finite element method (FEM) also by Rodriguez and Shammas [8].

Previous studies have revealed that critical failure modes in multilayer structures include interface delamination, micro cracking, and thermal fatigue [9,10]. Moreover, our coauthors have conducted some studies about the failures of micro multilayers, including thermal fatigue, cracking, and interface delamination, utilizing the Physics of Failure method [9,11,12]. However, little attention was given to the effect of the manufacturing process (both residual stresses and variation of design parameters) on the long-term reliability of multilayer structures. This factor is important for failure initiation and propagation in microscale devices because of the size and surface effects, as emphasised by the manufacturing manual and the reliability analysis reports of MEMSCAP^®^ actuators in the Polynoe program [13,14,15]. In addition, Huang and Zhang [16,17] analysed the effects of residual stresses on the development of bilateral (SiN_x_–Al) micro cantilevers based on IR (Infrared Range) detectors. Furthermore, Zhang and coworkers [18,19] evaluated the curvature induced by residual stresses (strains) and combined the effects of creep and stress relaxation in a thin film employing finite element simulations. Li et al. [20] studied the effect of residual stress on a SiN_x_ film and the corresponding improvement. In some works [21,22], the effects of parameter uncertainties on the function of MEMS were evaluated by stochastic finite element method (SFEM).

The objective of this research was to evaluate the effects of these manufacturing factors on thermomechanical deformation mechanisms as well as on failure onset and propagation in a multilayer structure. These variables are of vital importance for the design and analysis of micro actuators and for the improvement of their performance in the submicron scale. Since it is still difficult to detect damage in the internal structure of such devices by experimental testing approaches without destructing the specimens, the finite element (FE) analysis method based on a subdomain method was employed, which required the accurate assessment of stress and strain distributions to evaluate the failures. Hence, the verification of the subdomain model was granted by the adjusted geometry, material, and dynamic loadings that were obtained from the functional model of actuators, which was validated by experimental measures and specified analytical solutions.

## 2. MEMSCAP^®^ Actuator

### 2.1. Micro Actuator Presentation

**Figure 1:**
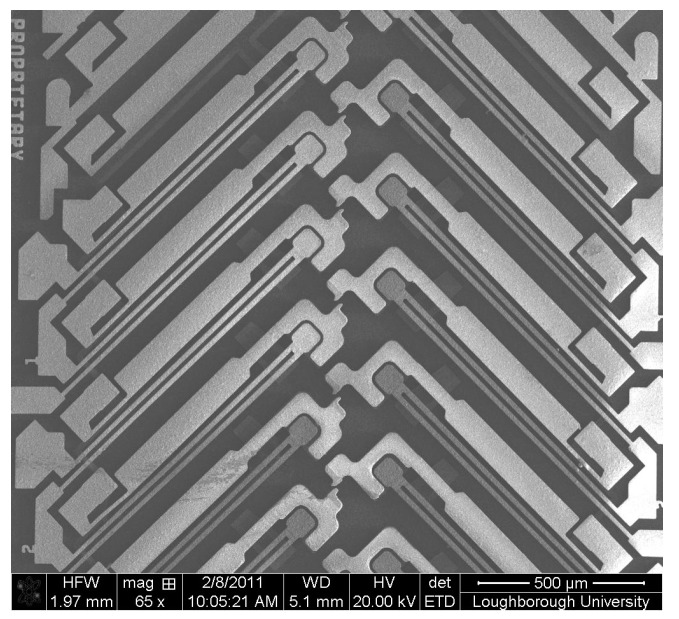
Micro actuator assembled in micro electromechanical systems (MEMS) devices. Reproduced with permission from [23].

**Figure 2:**
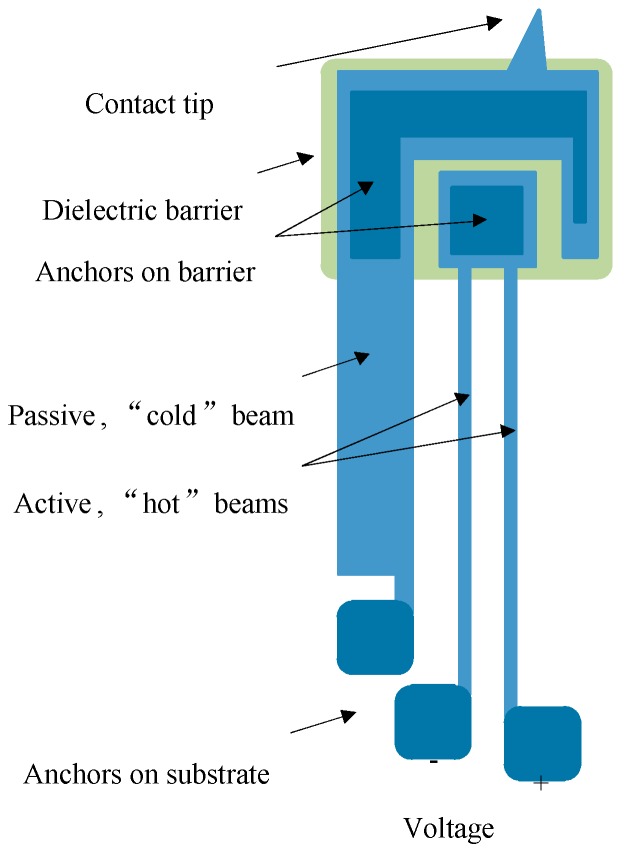
Two-hot-beam thermal actuator: A passive beam (“cold” arm) carries the electrical information, and two active beams (“hot” arms) are used for actuation; a dielectric barrier is between the passive and active beams. Reproduced with permission from [23].

The MEMSCAP^®^ actuator studied in this research is bistable and consumes no power in either the ON or the OFF position [12,13]. In order to make a latching switch, two actuators were utilized. Their assembly in MEMS devices is shown in Figure 1. The key component of an actuator is the U-shaped structure (“heatuator”) containing two thin “hot” beams and a wide “cold” beam (Figure 2). The “cold” beam, which was used to carry an electrical signal, was electrically isolated from the “hot” beams, actuating the switch. Different thermal expansions were used to achieve motion along the wafer.

**Figure 3:**
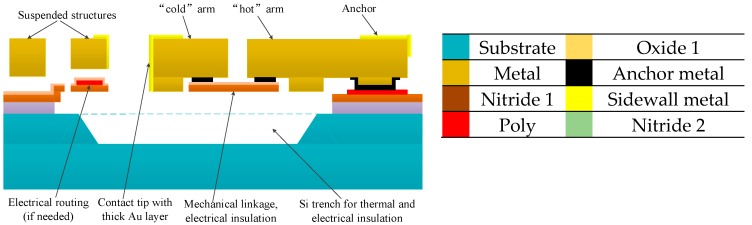
Cross section of the actuator and corresponding materials of the joint layers.

In the studied actuator, joint layers of different materials (from substrate to flexible metal cantilevers) were designed for better electrical continuity and thermal insulation. The well-established MetalMUMPs (Metal Multi User MEMS Processes) were used to manufacture this micro actuator [12]. Figure 3 represents a cross-sectional scheme of the multilayer cantilever micro actuator, where the various material layers are identified by a colour chart.

MetalMUMPs require 12 steps: (1) preparing a base wafer (N-silicon); (2) growing silicon oxide (Oxide 1) on the wafer surface and depositing a phosphosilicate glass layer as a sacrificial release layer; (3) coating the wafer with a UV(Ultraviolet)-sensitive photo-resist film, lithographically patterning it by exposing to UV light through the phosphosilicate glass layer, and then developing it; (4) adding a blanket layer of silicon nitride (Nitride 1); (5) doping and annealing a polysilicon film (Poly); (6) etching the second layer of silicon nitride (Nitride 2); (7) patterning and wet chemical etching the second sacrificial oxide layer (Oxide 2); (8) repeating the same procedure as in (7) to pattern the anchor metal; (9) plating the base layer with copper; (10) electroplating the nickel layer (Metal); (11) plating the gold layer (Sidewall metal) and removing the plating base from any exposed regions using wet chemical etching; (12) releasing and etching a Si trench.

### 2.2. Preliminary Analysis of the Multilayer Structure

Typical failure modes observed in the studied micro actuator include thermal fatigue, interface delamination, initiation and propagation of microcracks, material’s plastic deformation, and contact resistance degradation. These failure modes are closely linked to the heating of the flexible beams and the connected multilayer anchors. Different thermal expansions and geometries generate thermal stresses that vary along layer films and depend on the material properties, e.g., the Young’s moduli, the coefficients of thermal expansion, the thermal conductivity, etc. Also in [24], the scholars reached a similar conclusion: when manufacturing MEMS, the manufacturing process variations led to mismatches between the original designs and the final products.

Additionally, in the MetalMUMPs flow presented above, some nonuniformities could be observed on some wafers because of the insufficient quality of the final structure’s line width, which may have been caused by UV exposure or by the final thickness of the metal layers that could not be precisely defined in the electrodeposition process. Hence, it was decided to perform a process sensitivity study of the dimensions, critical for the selected modes of failure. 

For the mentioned manufacturing process, it also should be noticed that multilayer thin-film materials with various thicknesses, compositions, and deposition methods for each layer typically exhibit residual stresses. Occasionally, the presence of such stresses and strains can enhance or decrease the performance of the functional devices. To optimize the application of multilayer thin films, it is crucial to understand the nature of residual stresses and their effect on the reliability of a multilayer structure.

Therefore, these factors related to the control of the manufacturing processes interact with in-service loadings and bring uncertain effects on the onset and propagation of failures. This should be explored before fabrication in the failure analysis of the multilayer structure in micro actuators for a better design.

Undoubtedly, an experimental analysis such as transmission electron microscope (TEM) could be employed for estimating the mentioned problems in multilayer designs. However, its destructive character for the studied actuators limits its application in the failure analysis. Therefore, a numerical modelling method was employed and it is explained in the following sections, followed by a discussion of micromechanical modelling and simulation results.

## 3. Micromechanical Modelling

### 3.1. Geometrical and Material Property Characterization

**Figure 4:**
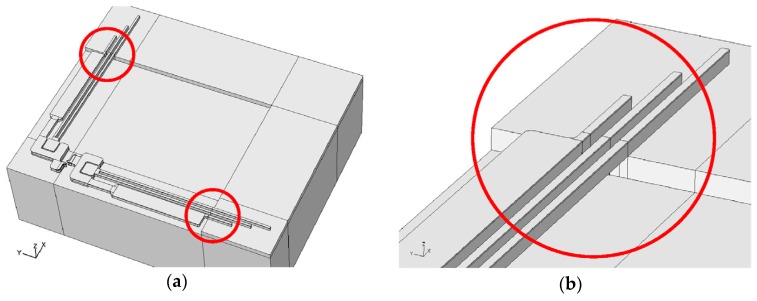
(**a**) 3D representation of the studied multilayer MEMSCAP^®^ switch and (**b**) detail of the cantilever beams.

**Figure 5:**
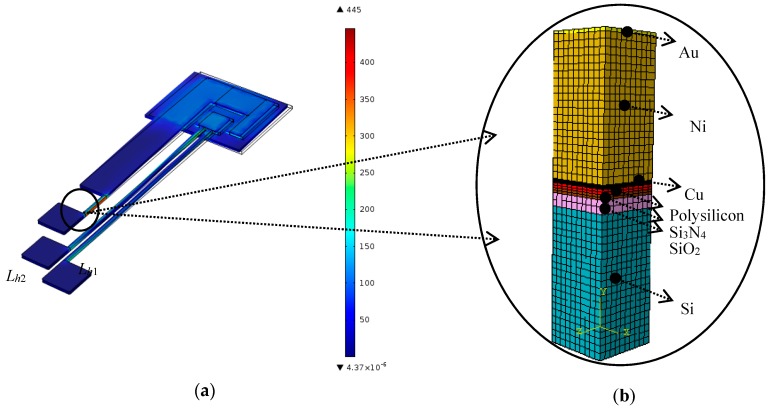
(**a**) Von mises stresses obtained with the verified function model of actuators and (**b**) subdomain multilayer structure of the micro actuator.

The numerical method begins with creating the geometry of the studied structure and analysing its material behaviours through the FE model using the COMSOL Multiphysics^®^ software (4.0, COMSOL, Stockholm, Sweden). The entire numerical model of the multilayer cantilever micro actuator is shown in Figure 4. Despite the very large number of elements in the mesh (more than 18,000,000) needed to detect the failure onset within the micro-scale multilayer structure, a fatal “out of memories” error occurred. Consequently, a subdomain method was chosen, in which a specific material structure and dynamic boundary conditions related to the powering up and down of the device were imported from a simplified multiphysics functional model (See Figure 5a).

**Figure 6:**
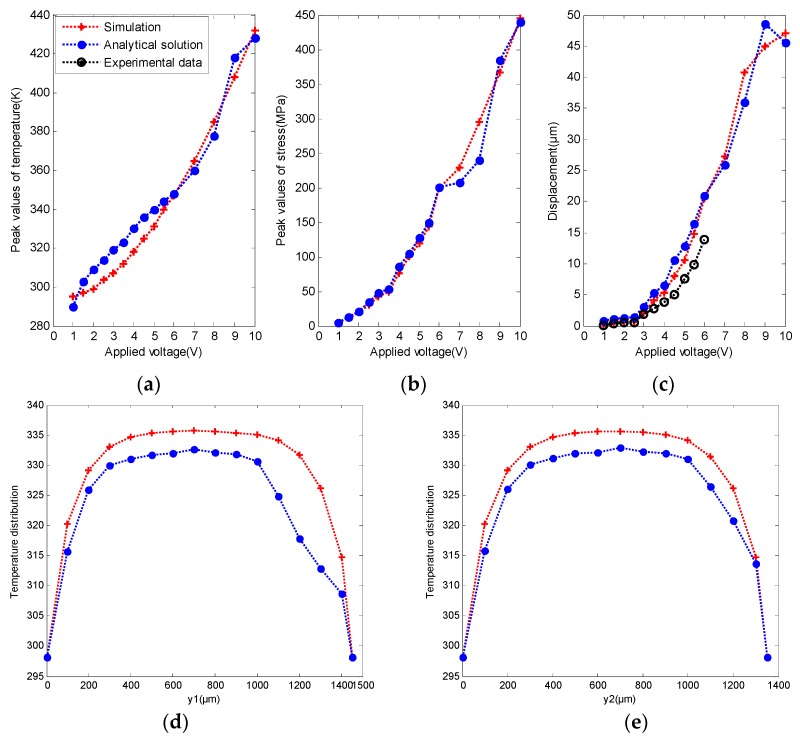
Verification of the simplified multiphysics simulations by analytical solutions and experimental data: (**a**) peak values of temperature with applied voltages (*U*) from 1 V to 10 V; (**b**) maximum stresses with applied voltages (*U*) from 1 V to 10 V; (**c**) displacement of the contact tip with applied voltages (*U*) from 1 V to 10 V; (**d**) transient temperature distribution of Lh1 at *U* = 5 V for type I boundary conditions; (**e**) transient temperature distribution of Lh2 at *U* = 5 V for type I boundary conditions. Reproduced with permission from [23].

To demonstrate the efficiency and feasibility of the proposed approach, two groups of data were employed to verify the multiphysics model using a set of figures: (i) comparisons of temperature, stress, and tip deflection value with validated analytical solutions and experimental data when the applied voltage (*U*) changed from 1 V to 10 V (as shown in Figure 6a–c) [23]; (ii) comparisons of the temperature distribution along the length of the “hot” arms, Lh1 and Lh2, with analytical solutions (as shown in Figure 6d,e) [23]. The power dissipated in a resistor is always proportional to *U*. Therefore, the temperature is also proportional to U, explaining the parabola observed in Figure 6a–c. As shown in Figure 6d,e, it was found that the temperature was largely uniform in the middle of the arms except near the ends, which is coincident with the conclusions in [25,26]. The results of the comparisons demonstrated that this multiphysics model was acceptable for the thermomechanical analysis to output boundary conditions for the subdomain model. 

Additionally, the multiphysics simulations arrived at the same conclusion as the practical observations, i.e., that the multilayer structure was one of the sites prone to failures in MEMSCAP^®^ actuators [12]. The geometry of the studied domain of the multilayer structure is shown in Figure 5b. 

This multilayer structure contains seven layers from the top to the bottom: gold, electroplated nickel, plating copper, polysilicon, silicon nitride, silicon oxide, and N-type silicon. This design allows a gradual electro transition from conductive to insulating properties for better performance. Some of the material properties in the metal layers are temperature-dependent, requiring the use of thermomechanical coupling. The thermal conductivity of gold varies sufficiently over the temperature range of interest to require the use of temperature-dependent values [27].

(1) $$k(T)=497T+41T^{2}-10.4T^{3}+0.56T^{4}-0.01T^{5},$$

The variation of the Young’s modulus *E* (GPa) and of the coefficient of thermal expansion $\alpha ({}^{\circ }C^{-1})$ with the temperature for the nickel layer can be calculated as [9,27]:

(2) E(T)=230×(1−0.000286T),

(3) $$\alpha (T)=13\times 10^{-6}\times (1+0.000343T),$$

The nickel layer demonstrated a linear kinematic hardening behaviour, and the kinematic strain hardening rate, *H_Ni_*, was experimentally evaluated to be 4 GPa (±2%) [9].

For the plating copper base, the material properties of the passivized copper films and the kinematic hardening model were utilized. The linear dependence of initial yield strength on temperature can be described by the following relationship [28]:

(4) $$\sigma_{y}=\sigma_{0}\cdot (1-T/T_{0}),$$

where σ_0_ and *T_0_* are reference constants. For passivized copper films, reliable results were obtained with σ_0_ = 305 MPa, *T_0_* = 1090 K. The kinematic strain hardening rate of copper is *H_Cu_* = 77 GPa [29].

All other properties are listed in Table 1 [9,27,28,29,30], where *Th* denotes the thickness of various layers, *ν* is the Poisson’s ratio, *Tan* represents the tangent modulus, and *H* is the kinematic strain hardening rate.

### 3.2. Dynamic Loadings for Failure Analyses

The concern over the reliability of multilayer structures arises from the large mismatch of thermal expansions between different materials in the through-thickness *y*-direction (as shown in Figure 5b). This mismatch generates a concentration of thermomechanical stresses when the multilayer structure is subjected to changes in temperature. The metal parts of the studied symmetric multilayer structure were connected to the flexible cantilevers of the actuators, hence, the displacement conditions should be consistent with their movement. Therefore, the dynamic loadings in the studied approach should include temperature changes and dynamic displacement boundary conditions.

(1) Thermal cooling and cycling

As already mentioned, residual stresses may be induced during the manufacturing processes because of the different thicknesses, compositions, and deposition methods of each layer. Theoretically, they can be either flow-induced (chain preferential orientations, freeze-off packing pressure, etc.) or thermally induced. In the studied thin films, the electroplating technique was used in MEMSCAP^®^ MetalMUMPs to form the multilayer structure. Thin films were deposited at elevated temperatures, followed by cooling to room temperature. Therefore, residual stresses could arise from the differential materials shrinkage as the temperature dropped from the process settings to the ambient postprocess conditions.

The electroplating process in MetalMUMPs takes place at relatively low temperatures (<140 °C), but other manufacturing processes might reach higher temperatures. As it was experimentally shown, in general, thermal cooling from temperatures about 60 °C–90 °C can cause yielding processes in the plating base (copper layer), which may result in nonrecovery expansions [9,10,11]. In order to overconstrain the structure, the boundary conditions applied were a combination of the kinematic constrain and the free boundary conditions. Thus, the following four cases of thermal cooling rate applied to the metal layers were analysed to examine the different effects on MetalMUMPs: Rate I, temperature difference *ΔT* from 30 °C to 0 °C, Rate II, *ΔT* from 60 °C to 0 °C, Rate III, *ΔT* from 80 °C to 0 °C, Rate IV, *ΔT* from 150 °C to 0 °C. The other materials were assumed to be stress-free. The total strain induced on the layers by thermal cooling could be derived from:

(5) $$d\varepsilon_{ij}=\delta_{ij}\alpha (T)\Delta T,$$

where $d\varepsilon_{ij}$ denotes the total strain increment and $\delta_{ij}$ is the Kronecker delta.

**Figure 7:**
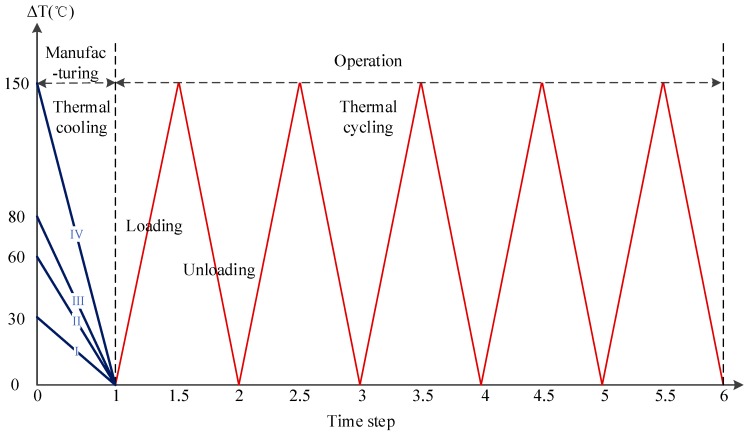
Thermal loadings include cooling and cycling process.

Several thermal cycles were repeated in all layers, as shown in Figure 7, until the change in magnitude of the plastic strain reached a steady-state value. The cyclic temperature increased from 0 °C to 150 °C, and then decreased from 150 °C to 0 °C.

(2) Dynamic displacement conditions

It was shown [9,10,11] that boundary axial and bending displacements represented critical configurations especially for the copper layer, where damage is expected because of the effect of a highly-localised stress–strain field. Therefore, these displacements should be simulated as close to the operation condition of the actuator (movement of flexible nickel cantilevers) as possible.

**Figure 8:**
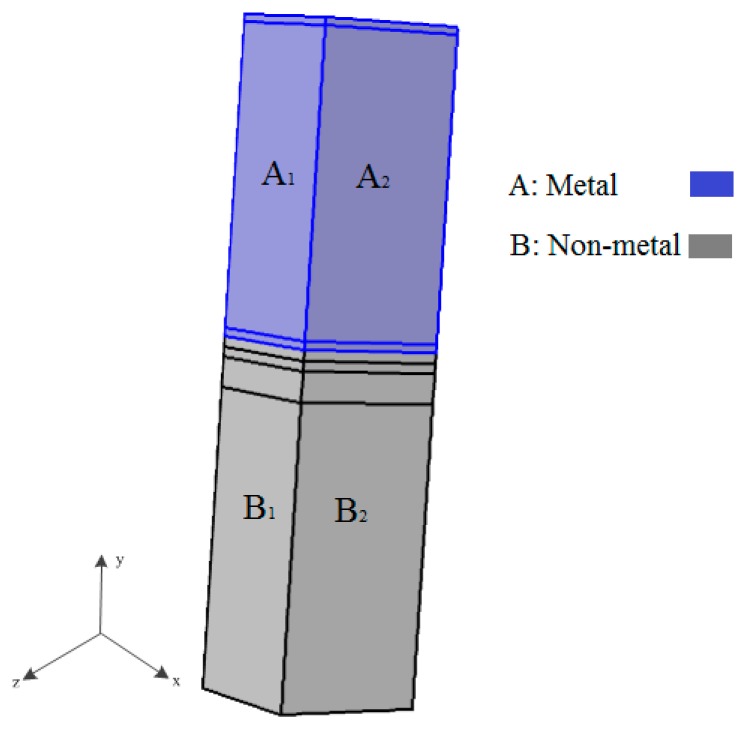
Subdomain configuration.

**Figure 9:**
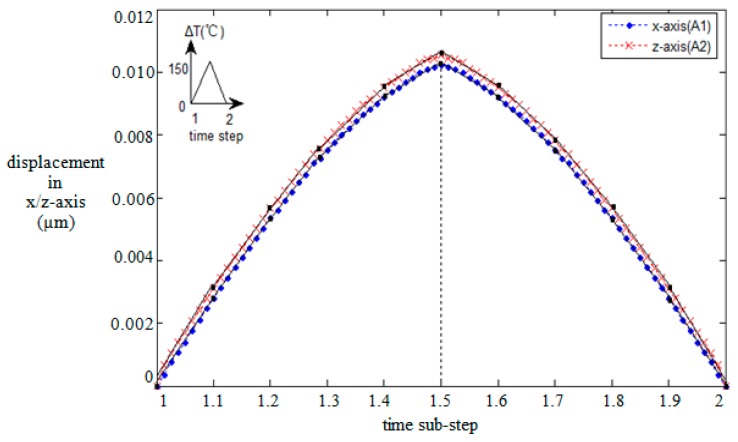
Dynamic displacement conditions along the faces A1 and A2 in time steps; the temperature is shown in the inset.

The subdomain configuration of multilayers is shown in Figure 8, where metal layers are shown in blue and nonmetal layers in grey. The bottom surface of the structure at *y* = 0 was fully constrained in *x*-, *y*-, and *z*-axis. The symmetry boundary conditions were applied in the planes at *x* = 0 and *z* = 0. The nonmetal faces B1 and B2 were assumed to be fixed substrates, thus the displacement along the *x*-, *y*-, and *z*-axis were constrained. The remaining surfaces A1 and A2 underwent dynamic displacement conditions caused by the thermo-induced mechanical deformation of the flexure beams (See Figure 5a). Such loading conditions were obtained by the multiphysics functional model, shown in Figure 9 [23]. The main task of this research was to identify a different combination of cooling and cycling thermal loadings and boundary conditions which might lead to damage (e.g., interfacial delamination, micro cracking, plastic deformation, and thermal fatigue) within the multilayer structure.

## 4. Results and Discussion

The principal factors to consider with respect to failure mechanisms of a multilayer structure are: (1) stress concentration resulting in the amplification of local stresses and strain because of thermal changes; (2) thermal fatigue caused by in-service thermal cycling; (3) residual stresses and predeformation of thin films caused by material cooling and the packaging process; and (4) the degree of local parameter variations at failure onsets. The seven-layer structure was meshed with the 187,425 hexahedral and quadrilateral elements of the vulnerable layer (copper layer) described with a finer mesh.

### 4.1. Stress Concentration and Strain Amplification under Thermal Transients

**Figure 10:**
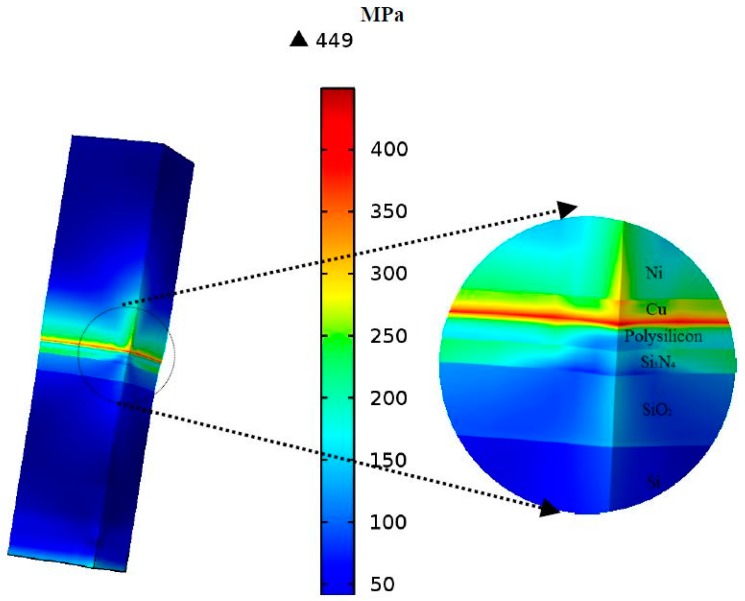
Distribution of von Mises stress in the multilayer structure.

**Figure 11:**
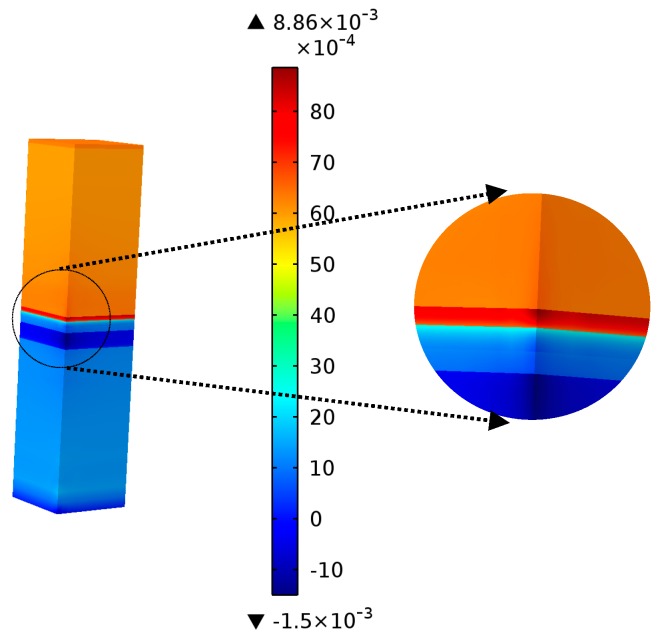
Distribution of plastic strain in the multilayer structure.

The stress and strain distributions take a significant part in defining the failure behaviours in MEMS devices, especially mechanically-induced problems. In a case used as a reference, the initial condition of the developed model did not include residual stresses in each layer. After several thermal cycles for the powering of the device, von Mises stresses and the elastic–plastic strain in the multilayer structure at *ΔT* = 150 °C of the last cycle were determined and are shown in Figure 10 and Figure 11, respectively. Yielding and plastic deformation were detected in the three layers (copper, nitride, and gold). The copper plating base was exposed to a severe stress concentration at the interface with the polysilicon, and to strain amplification at the interface with nitride. The high interfacial stresses generated the onset of initial yielding and a probable, successive interfacial delamination and micro cracking.

**Figure 12:**
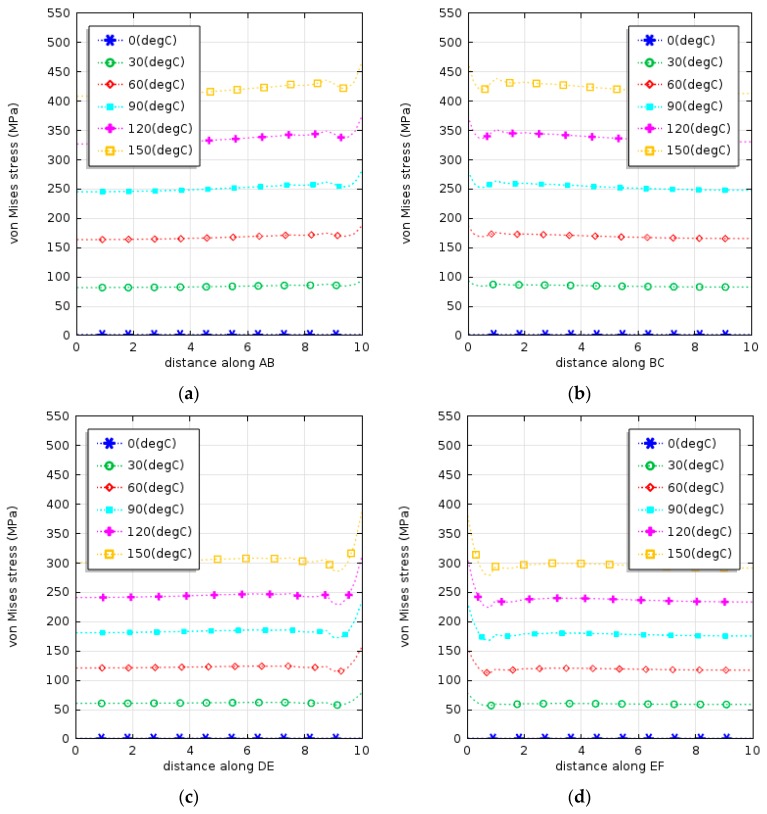

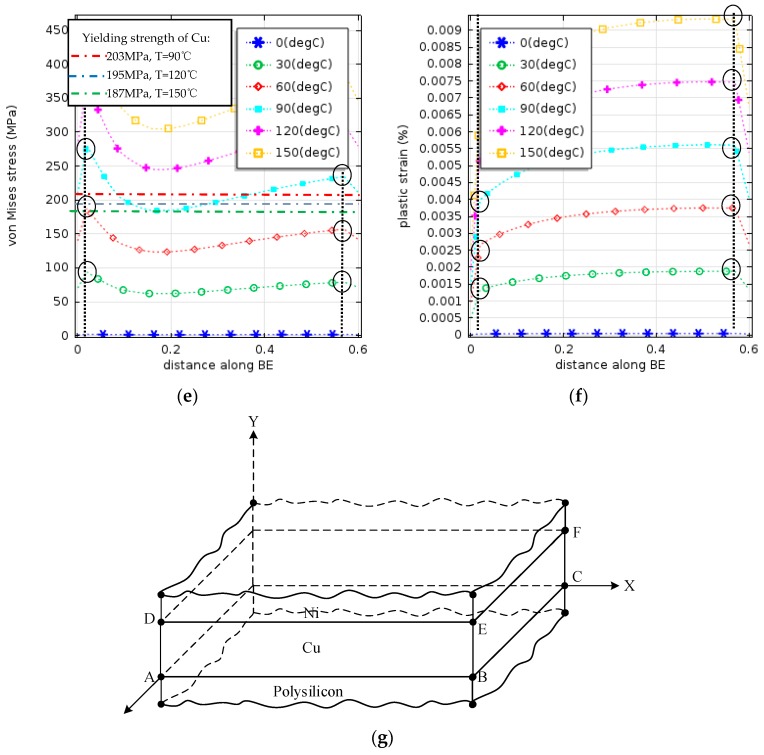
Distributions of von Mises stresses and plastic strains during the first half-cycle and their locations in the structure (**g**): along the lines AB (**a**); BC (**b**); DE (**c**); EF (**d**); BE (**e**,**f**).

The character of the evolution of von Mises stresses with thermal changes along the edge of Cu–polysilicon in the last half cycle from 0 °C to 150 °C is shown in Figure 12a,b. As expected, the highest stress concentration was located at the interface centre corresponding to the thin layers of copper and polysilicon, and reached up to 463 MPa (when the temperature change *ΔT* was 150 °C). It was shown that the applied loading and boundary conditions are of crucial importance for the yielding onset. Compared with that at the interface of copper and nitride, the general behaviour of the copper layer was characterized by both thermoelastic and plastic regimes, as shown in Figure 12c,d. Similar results were found by our research partners within the Polynoe program [12,13].

Additionally, the distribution of stress and plastic strain along the central edge of the copper layer from the polysilicon to the nitride layer are shown in Figure 12e,f. When the temperature change exceeded 90 °C, the corner part of the copper layer near the polysilicon layer could transit into the yielding process and, when the temperature change approached 120 °C, it totally underwent plastic deformation. In this loading condition, the multilayer actuator experienced a shear force at the interface of the different layers, while actuated. Consequently, delamination could occur after a long-term operation, decreasing the actuator's lifetime.

The results of the simulation, presented in Figure 12e,f, also demonstrated the presence of a singular point at the interface between different layers. This can be explained by the fact that the layers of different materials with varying coefficients of thermal expansions were assumed to be perfectly bonded together at their interfaces; consequently, the distorted shape was different from the ideal configuration.

### 4.2. Estimation of the Thermal Fatigue Lifetime

Since, when in service, the power was repeatedly switched on and off, the components were subjected to thermal cycling. Our stress and strain analysis showed that the most critical area was the thin copper layer. In particular, the maximum stress was concentrated at the interface with the polysilicon layer, and the maximum strain at the interface with nitride. These thermal and mechanical cycles and their coupling can result in the variation of the grain size (because of annealing in hottest regions), grain boundaries, and surface roughness, and they can accelerate the growth of micro- and nanoflaws in the structure. Ultimately, the progressive and localized structural damage can be caused by thermomechanical fatigue.

**Figure 13:**
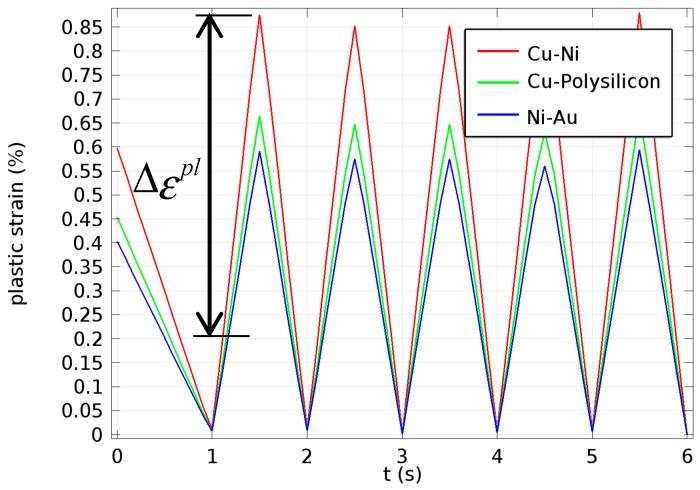
Plastic strain changes with time for three different interfaces: copper–nitride, copper–polysilicon and nitride–gold.

To estimate the thermal fatigue lifetime of the multilayer structure, the strain-based fatigue properties were introduced in the Coffin–Manson relationship:

(6) $$\frac{{\Delta \varepsilon }^{pl}}{2}=\varepsilon {\left(2N_{f}\right)}^{c},$$

where ${\Delta \varepsilon }^{pl}$ is the plastic strain amplitude (shown in Figure 13), *c* is known as the fatigue ductility exponent that, in general, varies from −0.5 to −0.7, *N_f_* is the fatigue life (in cycles), and *ε* denotes the fatigue ductility coefficient.

At the present stage of this research, five cycles were simulated to compute the equivalent plastic strain, ${\Delta \bar{\varepsilon }}^{pl}$:

(7) $${\Delta \bar{\varepsilon }}^{pl}={\Delta \bar{\varepsilon }}^{pl}{|}_{0}+\int_{0}^{t}{\Delta \dot{\bar{\varepsilon }}}^{pl}dt,$$

where ${\Delta \bar{\varepsilon }}^{pl}{|}_{0}$ denotes the initial equivalent plastic strain, and the classical metal plasticity (von Mises):

(8) $${\Delta \dot{\bar{\varepsilon }}}^{pl}=\sqrt{\frac{2}{3}{\dot{\varepsilon }}^{pl}:{\dot{\varepsilon }}^{pl}},$$

where ${\dot{\varepsilon }}^{pl}$ represents the plastic strain rate tensor. The plastic magnitude ${\Delta \varepsilon }^{pl}$ is defined by

(9) $${\Delta \varepsilon }^{pl}=\sqrt{\frac{2}{3}\varepsilon^{pl}:\varepsilon^{pl}},$$

Thus, the thermal fatigue lifetime computed by employing the results of simulations was equal to 1819 for *c* = −0.5, and to 174 for *c* = −0.7 for the reference case, without accounting for residual stresses. These values were compared with those for cases with residual stresses and variation of design parameters, as shown below.

### 4.3. Effects of Different Levels of Residual Stresses on the Long-Term Reliability of the Multilayer Structure

The studied multilayer structure was subjected to thermal-induced residual stresses related to its manufacturing process. Different thermomechanical parameters (e.g., different cooling rates), as the material solidifies from the mold wall to the centre, would produce various levels of residual stresses. In addition, from the analysis of stress and strain concentrations, it was found that at about 90 °C the yielding process started in the copper layer. Therefore, to fully investigate the effects of different regimes of postmanufacturing cooling, five cases were designed (See Figure 7): (i) without residual stresses, i.e., the reference group, (ii) Rate I, (iii) Rate II, (iv) Rate III, (v) Rate IV.

**Figure 14:**
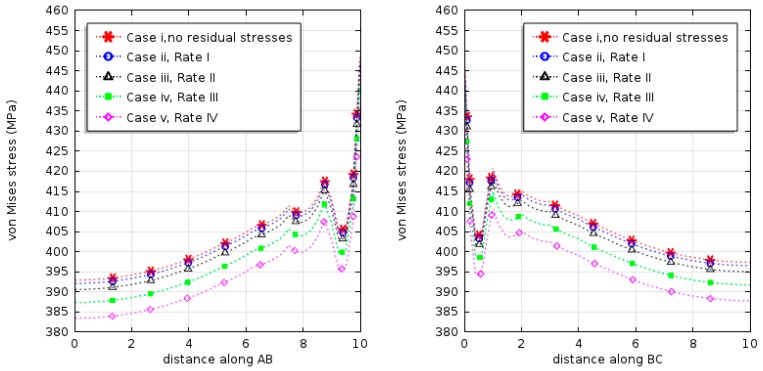
Von mises stress concentration at the copper–polysilicon interfaces at different cooling rate.

The simulation results for maximum levels of von Mises stress concentration along the copper and polysilicon interface for the five cases are shown in Figure 14; Table 2 presents a comparison of maximum strains and thermal fatigue lifetime in these cases.

From the analysis of the results shown in Figure 14, it is clear that material shrinkage during injection molding can be conveniently described by a typical state of stress characterised by compression in the multilayers balanced by expansions in the following loadings of thermal cycling. Residual stresses caused by cooling in the manufacturing process would help decrease stress concentration in the layers; cooling from higher temperatures was not found to be more beneficial. The analysis of the maximum plastic strain and fatigue lifetime shown in Table 2 demonstrated that an appropriate cooling regime did not significantly affect the plastic deformation and thermal fatigue. However, for cases with initial temperatures exceeding 80 °C, more plastic deformation accumulates in the thermal cycling, greatly reducing the lifetime of the multilayer structure.

Thus, when changing the state of the actuator between the “on” and “off” positions, appropriate residual stresses caused by thermal cooling could play a positive role in the actuator's thermal expansion. However, if the residual stresses and strains are beyond the elastic limit of the copper layer, they may accelerate the actuator's plastic deformation and affect detrimentally its reliability.

### 4.4. Effects of the Variation of Geometrical and Material Parameters on the Long-Term Reliability of the Multilayer Structure

The complex and highly variable nature of the microactuator’s manufacturing processes and of the electroplated deposition makes it difficult to empirically assess the influence of the involved parameters on the device performance. Thus, in this research, the FEM method was employed for this purpose. Both geometry and material variations could be examined with high levels of control and efficiency.

The results of the numerical analysis mentioned before demonstrated that the copper layer was the vulnerable area, presenting high concentrations of stresses and plastic strain. This was also supported by the experimental findings that both interface delamination and micro cracking arose in the copper layer. Thus, variations of the design parameters for the copper layer were considered to assess their effects on the fatigue behaviour of the studied multilayer structure under thermal cycling. Four cases of variations were considered, as listed in Table 3. In each case, only one of four parameters, i.e., the Young’s modulus, the coefficient of thermal expansion, the thermal conductivity, or the thickness of the copper layer was changed, while the reference values for the others were retained. Therefore, in case (a) there were five groups of data corresponding to different values of the Young’s modulus of copper; similarly, each of the other cases had five groups of data corresponding to different values of one of the other parameters, as shown in Table 3.

Finite element simulations of all 20 sets of parameters for the chosen four cases were used for a critical evaluation of the influence of significant design and material parameters on the fatigue life of the vulnerable parts in the micro actuator multilayer structure. The obtained maximum magnitudes of its plastic strain and the estimates of the thermal fatigue lifetime for the four cases are shown in Table 4. The bold font is used to highlight the reference set of parameters.

The analysis of the obtained results indicated that the most sensitive parameters to fatigue lifetime were: (1) the mismatch in thermal expansion between the copper and nickel layers (as shown in Case b) and (2) the thickness difference between the copper and polysilicon layers (as shown in Case d), which significantly decreased the stress concentrations. Thus, the most significant improvement to fatigue lifetime could be obtained by balancing these parameters. A ratio of 1:1 of these parameters appeared to enhance the life of the actuator, as compared with the reference groups. No significant change in the fatigue life was observed for other variations. These results also indicate that improved reliability may be obtained at the cost of performance. The designers of micro actuators should consider a balanced relationship between performance and reliability.

## 5. Conclusions

The long-term reliability of the MEMSCAP^®^ actuators can be greatly reduced because of failures of their multilayer structure wherein large stress concentration and delamination can emerge. Thus, it is necessary to predict the failure behaviours of the multilayer structures and investigate the effects of both the manufacturing process and the in-service On–Off operation loadings. Finite element simulations based on the subdomain method were employed in this research. To guarantee the validity of the numerical approach, dynamic displacement loading was adjusted employing the practical function model of micro actuator, validated with the experimental data and analytical solutions.

The obtained simulation results demonstrated that the maximum local stresses concentrate along the copper–polysilicon interface and the maximum strains were found along the copper–nitride interface. Both factors can result in potential failures, such as yielding or creep of materials, interface delamination, micro cracking, and thermal fatigue, as it was found by studies within the POLYNOE program on the same device [13,14,15]. 

Additionally, this research could provide important insights into the effects of the manufacturing processes on the performance of the actuator under conditions of thermal fatigue observed in the multilayer structure:

(i) the manufacturing processes could produce beneficial residual stresses at a specific range of cooling rates, lower than 90 °C;
(ii) sensitively adjusting the level of the coefficient of thermal expansion of copper and selecting a suitable thickness of the copper layer could help improve the reliability of the multilayer structure. These changes, though, should be balanced with the actuator's performance in terms of electrical and thermal continuity.

## Figures and Tables

**Table 1:** Material and geometrical properties of the multilayer structure.

Materials	Colour	$\begin{array}{c} Th\\ \left(\mu m\right) \end{array}$	$\begin{array}{c} E\\ \left(\mathrm{GPa}\right) \end{array}$	ν	$\begin{array}{c} \alpha (T)\\ \left({\times 10}^{-6}{}^{\circ }C^{-1}\right) \end{array}$	$\begin{array}{c} \sigma_{y}/\sigma_{ult}\\ \left(\mathrm{MPa}\right) \end{array}$	$\begin{array}{c} Tan\\ \left(\mathrm{MPa}\right) \end{array}$	$\begin{array}{c} H\\ \left(\mathrm{GPa}\right) \end{array}$	$\begin{array}{c} k\\ \left(W/(m\cdot K)\right) \end{array}$
Si	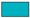	20	130	0.28	3.1	3790	-	-	130
SiO_2_	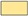	2	71.4	0.16	0.52	486	-	-	1.4
Si_3_N_4_	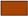	0.65	260	0.25	3.2	460	-	-	20
Polysilicon	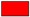	0.7	160	0.23	2.6	1200	-	-	78
Cu	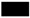	0.55	110	0.3	17	Equation (4)	480	77	400
Ni	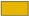	20	Equation (2)	0.31	Equation (3)	270	4000	4	90.7
Au	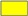	0.5	75	0.42	14.2	100	-	-	Equation (1)

**Table 2:** Estimation of fatigue lifetime for different cooling rates.

Case	Residual Stresses	Maximum Plastic Strain $\Delta \varepsilon^{pl}$(%)	Parameter *c*	Fatigue Life *N_f_*
i	None	0.663	−0.5	1819
			−0.7	174
ii	Rate I	0.655	−0.5	1864
			−0.7	177
iii	Rate II	0. 660	−0.5	1836
			−0.7	175
**iv**	**Rate III**	**0.778**	**−0.5**	**1321**
			**−0.7**	**139**
v	Rate IV	0.98	−0.5	832
			−0.7	100

**Table 3:** Parameters of cases used to study the effect of variations of geometrical and material parameters.

Case	Young’s Modulus *E* (GPa)	Coefficient of Thermal ExpansionCTE $\alpha \;({\times 10}^{-6}{{}^{\circ}C}^{-1})$	Thermal Conductivity *k* (W/(m·K))	Thickness of Copper: $\begin{array}{c} Th\;(\mu m) \end{array}$
a	{70, 90, 110, 130, 150}	17	400	0.55
b	110	{13, 15, 17, 19, 21}	400	0.55
c	110	17	{200, 300, 400, 500, 600}	0.55
d	110	17	400	{0.35, 0.45, 0.55, 0.65, 0.75}

**Table 4:** Estimates of fatigue life.

Case	Maximum Plastic Strain $\Delta \varepsilon^{pl}$ (%)	Parameter *c*	Fatigue Life *N_f_*
a	{0.82, 0.75, **0.735**, 0.68, 0.66}	−0.5	{1189, 1422, **1480**, 1730, 1836}
		−0.7	{129, 146, **150**, 168, 175}
b	{0.591, 0.661, **0.733**, 0.8, 0.8}	−0.5	{2290, 1830, **1488**, 1250, 1250}
		−0.7	{206, 175, **151**, 133, 133}
c	{0.733, 0.733, **0.711**, 0.733, 0.733}	−0.5	{1488, 1488, 1582, 1488, 1488}
		−0.7	{151, 151, 158, 151, 151}
d	{1.02, 0.93, **0.733**, 0.6, 0.55}	−0.5	{768, 924, **1488**, 2222, 2283}
		−0.7	{94, 107, 151, 201, 264}
